# Teleintervention’s effects on breastfeeding in low-income women in high income countries: a systematic review and meta-analysis

**DOI:** 10.1186/s13006-024-00631-2

**Published:** 2024-04-13

**Authors:** Madeleine Corkery-Hayward, Mohammad Talaei

**Affiliations:** 1 Barts and The London School of Medicine and Dentistry, Queen Mary University of London, London, UK; 2 Wolfson Institute of Population Health, Barts and The London School of Medicine and Dentistry, Queen Mary University of London, London, UK; 3 Barts and The London Medical School, Queen Mary University of London, London, E1 2AD UK

## Abstract

**Background:**

Many mothers in high-income countries (HIC) do not breastfeed to the World Health Organisation’s recommendation of two years. This is particularly true for low-income women (LIW). They often face additional socio-structural barriers that encourage early discontinuation and are inadequately supported by current healthcare interventions. Teleinterventions are flexible and widely used following the global pandemic and increase maternal autonomy over intervention delivery. They show promise in improving other maternal conditions in LIW, including postpartum depression. Teleinterventions can increase breastfeeding rates in the wider maternal population, however their efficacy for this underserved population has not yet been systematically assessed. This meta-analysis aimed to identify if teleinterventions increase ‘exclusive’ or ‘any’ breastfeeding by LIW in HIC at 1-, 3–4, and 6-months postpartum.

**Methods:**

We searched five online databases for randomised controlled trials assessing breastfeeding teleinterventions for LIW in HIC. Risk ratios (RR) were used to calculate the average effect of teleinterventions on ‘any’ and ‘exclusive’ breastfeeding at at 1-, 3–4, and 6-months postpartum using random effects meta-analysis. Study bias was assessed using the Revised Cochrane risk-of-bias tool for randomised trials (RoB2), and outcome quality was evaluated against GRADE criteria.

**Results:**

Nine studies met inclusion criteria: six providing telephone calls, two text messages and one an online support group. All the studies were conducted in the United States, with small sample sizes and a high risk of bias. Pooled results indicate teleinterventions modestly increase ‘any’ and ‘exclusive’ breastfeeding at all time points, with a statistically significant increase in ‘exclusive’ breastfeeding after 3–4 months (RR 1.12, 95% CI [1.00,1.25]). At 3–4 months teleinterventions providing peer support were more effective than educational teleinterventions at promoting any and exclusive breastfeeding. Evidence for all outcomes were rated ‘low’ or ‘very low’ quality using the GRADE tool, mainly due to high attrition and low power.

**Conclusions:**

Despite insufficient high-quality research into breastfeeding teleinterventions for LIW, our results suggest teleinterventions may improve exclusive and any breastfeeding. Given breastfeeding is particularly low in LIW population from HIC, our findings are promising and require further exploration by larger, methodologically sound trials in other HIC.

**Supplementary Information:**

The online version contains supplementary material available at 10.1186/s13006-024-00631-2.

## Background

Increasing the number of women who breastfeed is a global public health priority. Rates persistently vary between countries and are often the lowest in high-income countries (HIC). The most recent data from the United Kingdom (UK) shows that as few as 1% of infants exclusively breastfeed up to six months postpartum in 2020, and less than half breastfeed at all after eight weeks [1]. Within HIC, breastfeeding often reflects wider health inequities; in the UK and the United States (US), mothers in the lowest deprivation decile or lowest income (low-income women, LIW) are least likely to start breastfeeding and have the highest risk of early cessation [2].

Remote technology-based care (teleinterventions) may be the solution. Teleinterventions are broadly defined as any remotely delivered technology-based care, encompassing a wide range of delivery modes such as phone calls, internet groups, and smartphone applications [3]. Their flexibility and ease of access have led to them being increasingly adopted by multiple disciplines in the wake of the global pandemic. They have now been proven to effectively promote general health in LIW and improve other maternal conditions, including postpartum depression [4–7]. Emerging evidence indicates they may also successfully promote breastfeeding initiation and duration in the wider maternal population, where traditional interventions have failed [6, 8–10].

Promisingly, studies in the US demonstrate a widespread acceptability of health applications by LIW, highlighting their ability to increase engagement and perceived support [11]. Therefore, teleinterventions may be particularly effective in promoting breastfeeding in low-income women in HIC.

Breastfeeding is an individual decision that influences and is influenced by multiple factors. Mothers in HIC are faced with multiple socio-structural barrier and a strong infant formula culture [12]. LIW are subject to the same problems as more affluent mothers but have fewer resources to overcome them [4, 13]. Global economic disruption has increased the proportion of mothers living in poverty [14]. Given the persistent socioeconomic disparities in breastfeeding in HIC, this has worrying implications for future infant feeding practices and population health.

Research from HIC report that LIW often have reduced community support and feel stigmatised by healthcare professionals over their infant feeding choices [13, 15]. This exacerbates additional structural barriers (such as increased childcare demands), and consequently many breastfeeding interventions are inaccessible for LIW [13, 15]. The COVID-19 pandemic highlighted this inequity; lower-income mothers in the UK were disproportionately affected and more likely to stop breastfeeding, with 70.3% attributing this to a lack of support [16].

To date, no reviews have focused the effect of teleinterventions on breastfeeding in LIW. This population faces additional sociostructural barriers and consequently many services which are effective in the wider population are not for LIW [4, 6]. Thus, the promising teleinterventions results in the general population do not necessarily hold true for LIW [4]. This study aimed to address this gap in the literature and determine if teleinterventions can effectively promote breastfeeding in LIW living in HIC.

## Methods

This systematic review followed the Preferred Reporting Items for Systematic Reviews and Meta-Analyses (PRISMA) guidelines and is registered with the PROSPERO register (2020: CRD42021278833) [17].

Breastfeeding initiation is a complex decision, heavily impacted by immediate postpartum support and the clinical environment [18]. Most mothers start breastfeeding but rapidly stop [1]. Therefore, the primary outcome of this review was exclusive breastfeeding at one, three-to-four, and six months - as breastfeeding challenges and cessation are most common in these periods [19]. Exclusive breastfeeding is the ideal, however any breastfeeding is beneficial, and therefore ‘any breastfeeding’ at the same timepoints was a secondary outcome.

### Search strategy

The Population Intervention Comparator Outcome (PICO) criteria was used to identify suitable keywords and medical searching heading (MeSH) terms (Additional File Table 1). Keywords included: low-income, Mobile heath/eHealth/ mHealth/telemedicine and Breastfeeding, (see Additional File for full search strategy).

**Table 1:** Characteristics of included studies

Study	Location	Participant Number	Implementation Period	Timepoints of Data Collected and Used by this Meta-Analysis	Study Breastfeeding Definitions	Main Intervention Delivery Mode	Main Intervention Component	Intervention
Bunik 2010	Single urban hospital in Colorado, US.	341 (I = 161, C = 180).	Postpartum (2w)	**AB** = 1 m, 3 m, 6 m	**AB =** ‘Any breastfeeding’, including ‘Predominant breastfeeding’ (< 4 oz formula daily)	TC	E	Daily educational calls from nurses using predetermined script addressing potential physical problems, benefits of breastfeeding, milk storage and alternative feeding and maternal health.
Efrat 2015	Five community health clinics in Los Angeles, US	289 (I = 146, C = 143)	Antenatal (four calls during third trimester) Postpartum (6 m)	**EB =** 1 m, 3 m, 6 m **AB** = 1 m, 3 m, 6 m	**EB** = ‘baby never received water, formula, folk remedies, or other foods’ since birth **AB** = At least once since birth, infant received water, formula, folk remedies or another food	TC	E	Four prenatal calls and seventeen postpartum calls by lactation educators.
Fiks 2017	Two obstetric clinics in Philadelphia, US	87 (I = 43, C = 44)	Antenatal (2 m) Postpartum (9 m)	**EB** = 6 m **AB** = 6 m	**EB =** Currently exclusively breastfeeding (undefined) **AB** = Ever breastfed (undefined)	Complex– multi-component interactive online group	C	Facilitated Facebook group of 7–13 mothers with online activities for 11 m, including weekly educational videos and psychologist led online support groups.
Martinez-Brockman 2018	Health centre, teaching hospital and two community agencies in Connecticut US	174 (I = 94, C = 80)	Antenatal (from 28w) Postpartum (3 m)	**EB** = 3 m **AB** = 3 m	**EB** = Consumption of only breastmilk **AB** = ‘Partial breastfeeding’ where infant received ‘breastmilk and formula but no solids. ‘	TM	E	Routine ‘Loving Support’ peer counselling alongside scheduled TM (< 160 characters).
Palacios 2018	4 WIC clinics in Hawaii (HI) and 2 in Puerto Rico (PR)	202 (I = 102, C = 200)	Antenatal and Postpartum Total duration = 4 m	**AB** = 3 m	**EB** = Consumption of only breastmilk **AB** = Partial breastfeeding (undefined).	TM	E	18 w of bidirectional weekly TM (35–50 words).
Pugh 2002	Urban hospitals, US	41 (I = 21, C = 20)	Postpartum (6 m)	**EB** = 3 m, 6 m **AB** = 1 m, 3 m, 6 m	**EB =** ‘Exclusive (undefined) **AB** = Nonexclusive breastfeeding (undefined)	TC	PS	Daily visits from a nurse whilst in hospital and three home visits (1,2 and 4w postpartum). Biweekly telephone support from a community-based peer counsellor for 8w, then weekly for four more months.
Pugh 2010	Two urban hospitals in United states	328 (I = 168, C = 160)	Postpartum (6 m)	**AB** = 3 m, 6 m	**AB** = ‘Infant receiving breast milk within the last 24 h’	TC	PS	Daily hospital visits by support team until discharge, followed by three 60 min home visits within a month. Minimum biweekly TC for 6 m by a peer counsellor and 24 h nurse access through telephone helpline.
Reeder 2014	Two rural and two urban clinics, Oregon, US	1948 separated into three arms (intense = 625, low intensity = 625, C = 635).	Antenatal (2 calls) Postpartum (low intensity = 2w, high intensity = 4 m)	**EB =** 1 m, 3 m, 6 m **AB** = 1 m, 3 m, 6 m	**EB** = Breastfeeding and not using formula. **AB =**Formula or solids introduced to child’s diet	TC	PS	Peer councillor support with two intervention intensities: Low intensity = 4 scheduled postpartum TC High intensity = low intensity plus 4 additional calls 1,2,3 and 4 m after delivery.
Srinivas 2015	Single urban antenatal clinic, US	120 (I = 50, C = 53, lost at 1 m = 17)	Postpartum (4 m)	**EB** = 1 m, 6 m **AB** = 1 m, 6 m	**EB =** ‘Only breastfeeding or breast milk feeding since birth’ **AB** = Infant started breastfeeding but not receiving exclusively breast milk.	TC	PS	Peer counselling mainly provided through phone calls. Contact scheduled once antenatally, within 5 days of delivery, weekly for 4w, biweekly between 4-12w and once at 4 m.

Key: I = intervention group, C = control group, w = weeks, m = months, AB = Any breastfeeding, EB = exclusive breastfeeding, TC = telephone calls, TM = text messages, E = education, PS = peer support, C = combined education and peer support

Six databases were selected based on an exploratory literature review: PubMed, EmBase, APA PsychInfo, Web of Science, and the Cochrane Child Health and Pregnancy and Childbirth databases. The search was run in February 2021 and repeated in April 2023 to ensure inclusion of more recent studies. For comprehensiveness, included studies’ bibliographies were also manually checked. The search strategy was initially peer reviewed and tested in PubMed and keywords then adapted where necessary.

### Eligibility criteria

Studies were included if they were RCTs conducted after 2000 in a HIC for a teleintervention initiated in the anti- or perinatal period with the primary or secondary aim to improve breastfeeding practices (Table 1).‘Low-income’ is a relative term with international variation [20]. Therefore, this review was guided by individual study’s definition and included those defining their population with these terms. We included studies that followed the WHO recommendation of four in person maternal support visits < 6 weeks of delivery, provided all other breastfeeding support was delivered remotely via teleinterventions [21, 22]..

Studies primarily concerned with adolescent or HIV positive women, pre-term births or mothers with complicated deliveries were excluded. These women face additional biosocial breastfeeding barriers and so are unrepresentative of the wider maternal population [18]. Two reviewers (MCH and MT) screened the resultant title and abstracts from each database against the eligibility criteria and any disagreements were discussed to reach a consensus. The rationale for excluding studies was recorded (Additional File Table 2).

### Quality assessment

The quality of evidence from eligible studies was assessed using the ‘Grading and Recommendations Assessment, Development, and Evaluation’ (GRADE) tool. This categorises research into four quality levels (high, moderate, low, and very low) [23]. Many studies may contribute to a single outcome and the outcome quality is set by the lowest rated contributing study. Bias was evaluated using the Revised Cochrane risk-of-bias tool for randomised trials (RoB2). RoB2 considers five bias domains (Additional File Table 3) and rates these as having a “low risk of bias,” “some concerns,” or “high risk of bias” [24]. The final study judgement is based on the lowest rated domain [24]. This was assessed by two reviewers and any disagreements discussed until a consensus was reached.

### Data collection and statistical analysis

Data were analysed using the Review Manager Version 5.4 (The Cochrane Collaboration) software. The number of women breastfeeding at one, three-to-four and six months was extracted, the data checked by two reviewers and then inputted to calculate the respective Risk Ratios (RR) [25, 26]. The pooled average effects were provided as RR with accompanying 95% confidence intervals (CI).

Pugh et al. (2002) presented breastfeeding changes in a line chart [27]. As raw data was unavailable, this was converted to numerical data using WebPlotDIgitalizer (online software recommended by Cochrane) [28]. The ‘random effects’ meta-analysis model and inverse-variance method was used to indicate the average effect of each teleintervention [25, 29].

Interstudy heterogeneity was assessed using the I^2^ statistic, with I^2^ > 30% indicating some heterogeneity, 30–60% moderate, and > 50% substantial heterogeneity [25]. Following Fu’s recommendation, when there were more than four studies with heterogeneity, a subgroup analysis was performed [30]. Subgroups were set as:

- Interventions delivering only education.
- Interventions providing peer support.

## Results

### Study selection

**Fig. 1:**
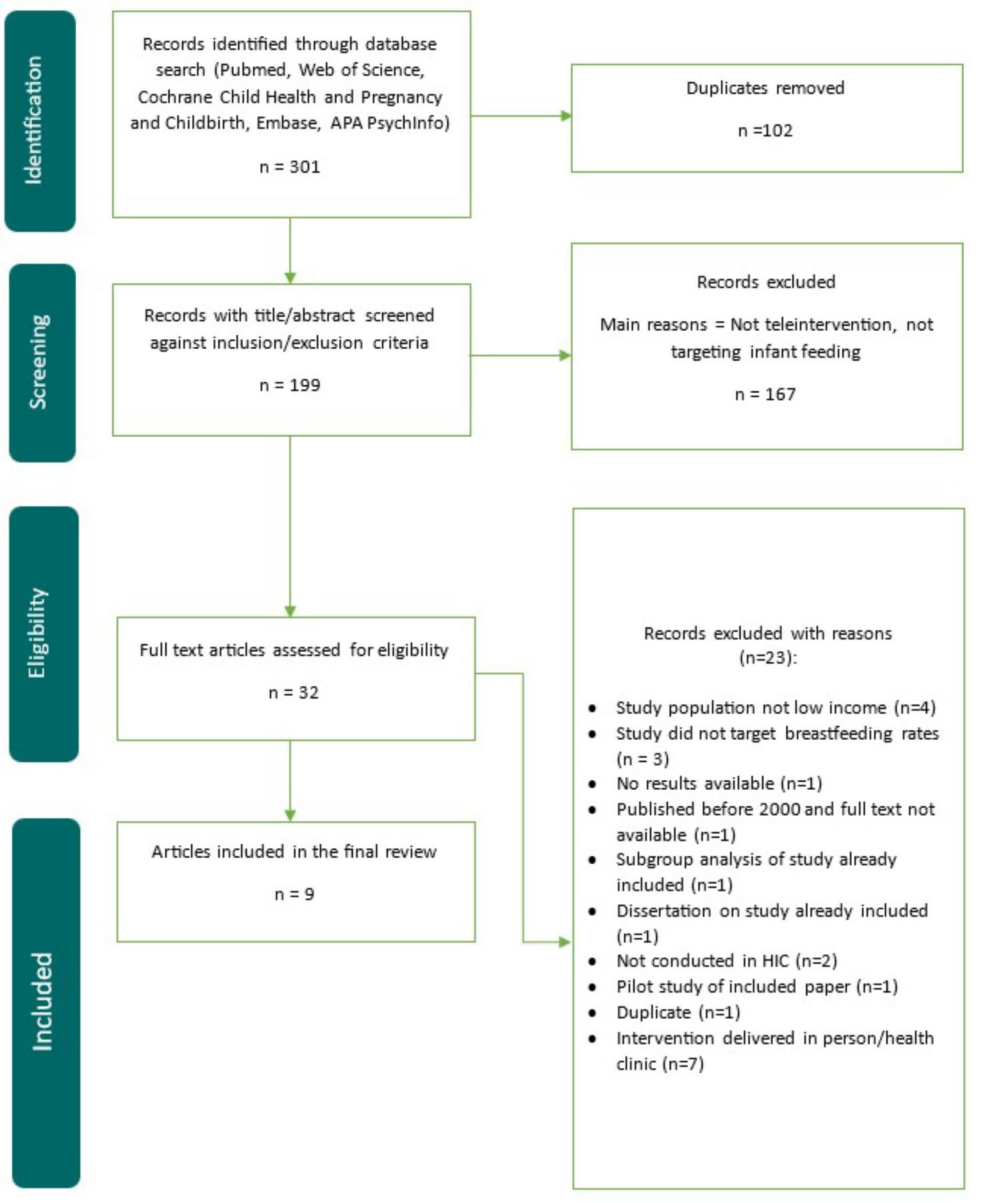
PRISMA flowchart of study selection process

An initial search across six databases identified 301 records (172 after duplicates removed). Title and abstract analysis excluded 140 studies, leaving 32 potential studies - narrowed to nine studies after reading full text. Most excluded studies did not focus specifically on LIW, used a teleintervention to facilitate in-person visits or provided teleinterventions to both the control and intervention group (for further details see Additional File Table 2). Study selection process is outlined in Fig. 1.

### Study characteristics

All the nine studies included in this review were conducted in the US between 2002 and 2020. Together they included 3522 mothers [27, 27, 31–38]. Most studies focused on ethnic minorities (Hispanic or African American), either by design [34, 38] or by virtue of area demographics [27, 31, 32, 39]. Palacios et al. covered mainly White and Hispanic mothers [35] and only one study had a majority of White mothers [33]. In all studies the mean maternal age was between 20 and 30 years, lower than that in general population in many HIC (30–34 years) [40].

Most studies did not collect data on factors known to affect breastfeeding (parity, delivery mode and previously breastfeeding), limiting results’ comparability [18]. Six studies did not record delivery method [27, 32, 33, 35, 37, 38], three did not include parity [27, 31, 33] and just three recorded previous breastfeeding experience [37–39]. Only one study recorded all the three factors [39]. Study characteristics are outlined in Table 1.

Most teleinterventions were phone based; six studies delivered breastfeeding support through telephone calls [27, 31, 33, 34, 38, 39] and two utilised text messages [35, 37]. Only Fik et al. assessed a complex web-based support group with online sessions, regular posts, and psychological support [32]. Of the nine included trials, four were delivered postpartum [27, 33, 34, 39], and the other five were conducted during both the antenatal and postpartum periods. The definition of ‘exclusive breastfeeding’ varied between studies and was not reported by two papers who instead recorded ‘any’ or ‘predominant’ breastfeeding [34, 39].

### Bias

**Fig. 2:**
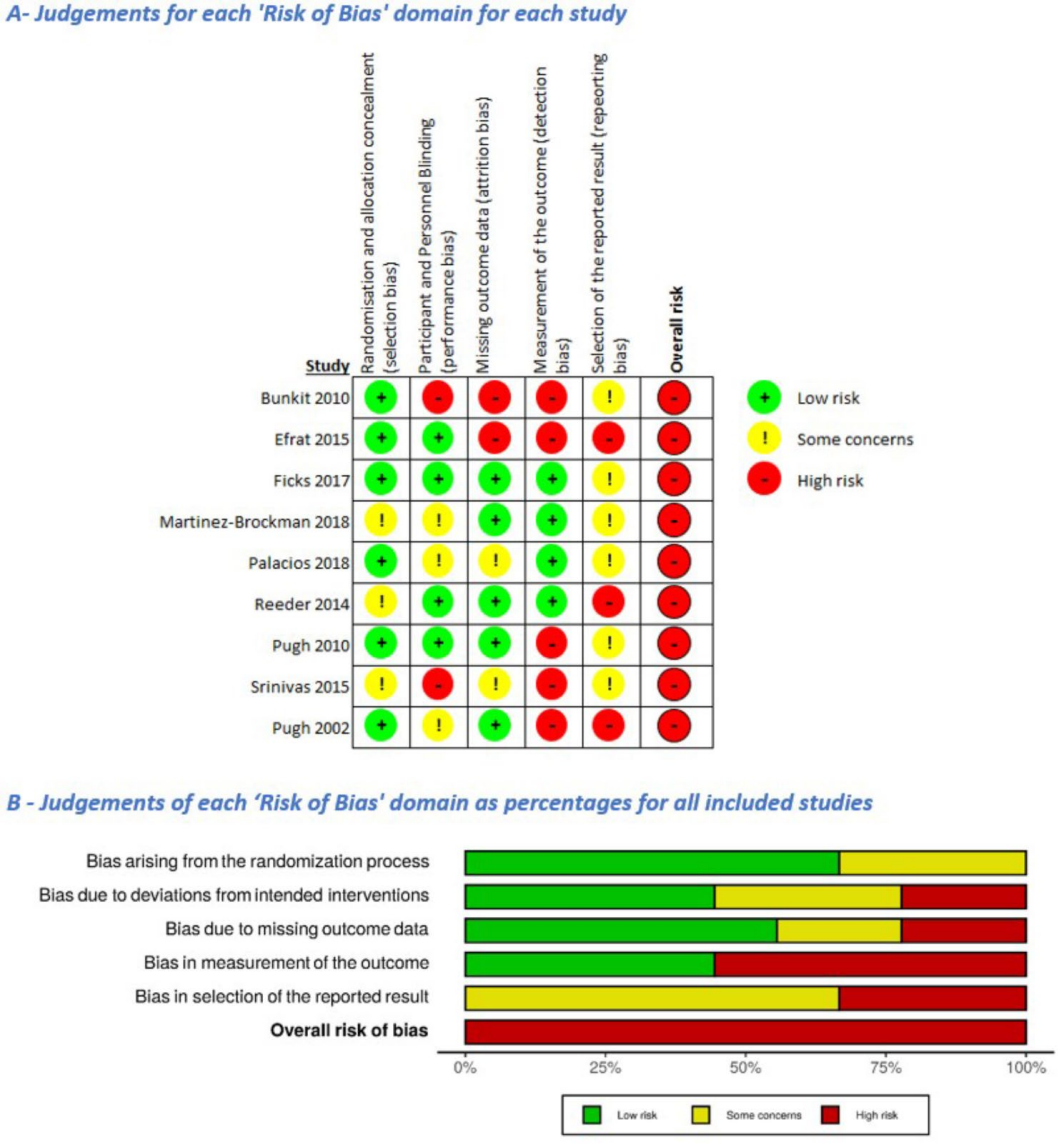
‘Risk of Bias‘ summary

All studies included in this review had a high risk of bias, represented in Fig. 2A and B (full rationale presented in Additional File Table 4). Three studies provided insufficient information on the allocation sequence generation and implementation, raising ‘slight concerns’ of selection bias [31, 33, 37]. Two studies were judged as having ‘serious concerns’ of performance bias as they did not specify if the data collector was an external agent (not the peer support worker) [27, 34]. Trial protocols for three studies were unavailable and no protocols included a full analysis plan [27, 33, 38]. The consequential lack of a pre-publication analysis plan raises concerns of reporting bias in all trials. Additionally, three studies had ‘serious concerns’ of selective reporting due to protocol deviations [31, 38] or insufficient analysis information [27]. Funnel plots were not used to assess publication bias, as they have a low predictive power with < 10 studies.

### Exclusive breastfeeding

**Fig. 3:**
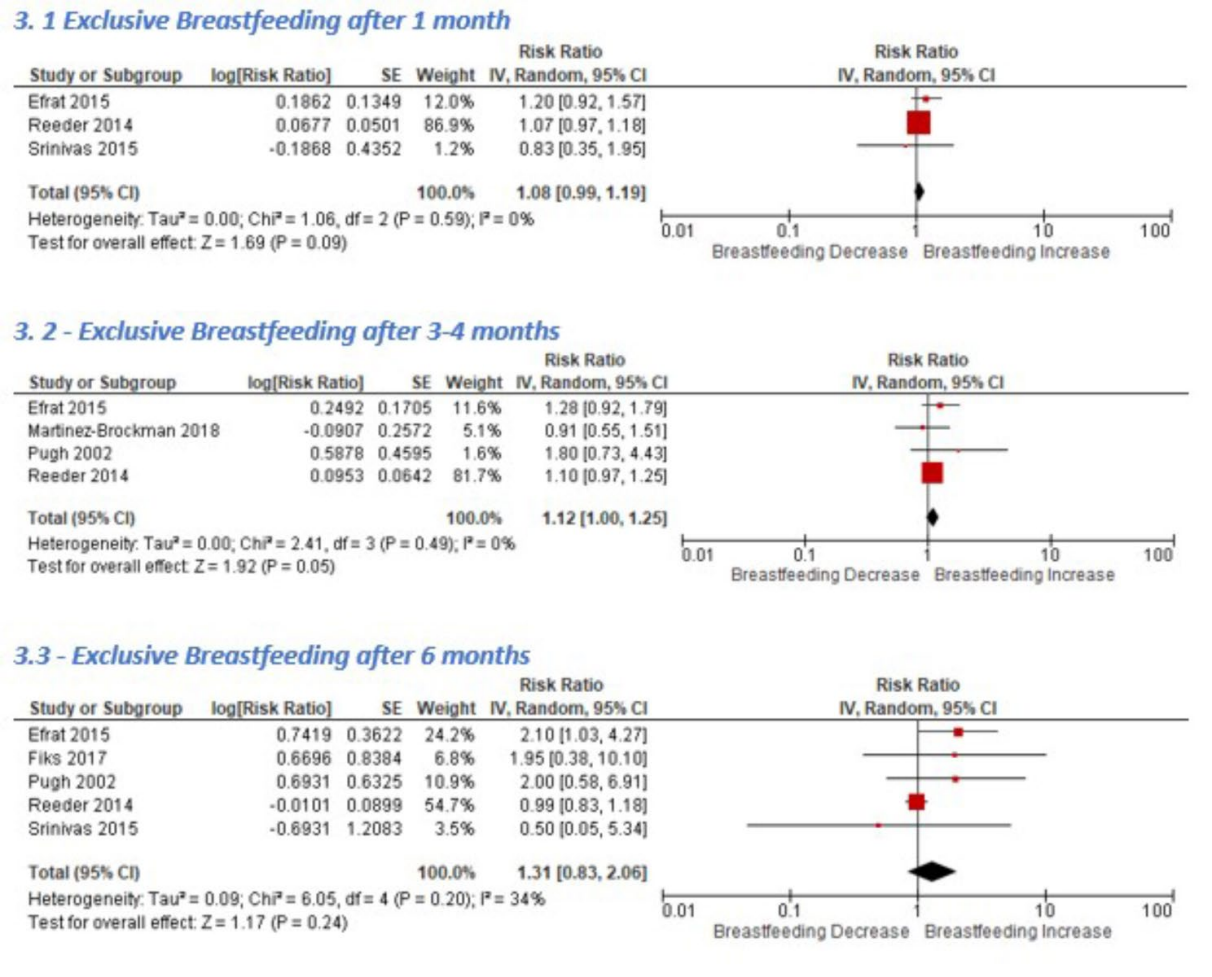
Effect of teleinterventions on exclusive breastfeeding

The number of mothers who exclusively breastfed for six months was only measured by five of the included studies [27, 31–33, 38]. Three studies recorded EBF at one month, all of which assessed supportive phone calls [31, 33, 38]. Two provided postpartum peer support and one delivered ante- and postnatal phone education from lactation educators [38]. The average effect from the pooled results indicates a modest breastfeeding increase, with borderline statistical significance (Fig. 3).

Four studies recorded EBF at 3–4 months, and their pooled results indicate teleinterventions may marginally increase EBF (Fig. 3). This effect was slightly attenuated following a sensitivity analysis which excluded Efrat et al.’s study due to the high risk of attrition bias (RR 1.10, 95% CI 0.97–1.24) [38].

Pooled results (Fig. 3) at 6 months show a beneficial but non-statistically significant effect on EBF, which almost disappeared in a sensitivity analysis excluding studies with particularly high attrition (RR 1.01, 95% CI 0.85–1.2) [33, 38].

### Any Breastfeeding

Definitions of ‘partial breastfeeding’ varied between studies [34, 38]. To standardise pooled results this meta-analysis used the subcategory that included all breastfeeding mothers (exclusive, any or partial) from each study.

**Fig. 4:**
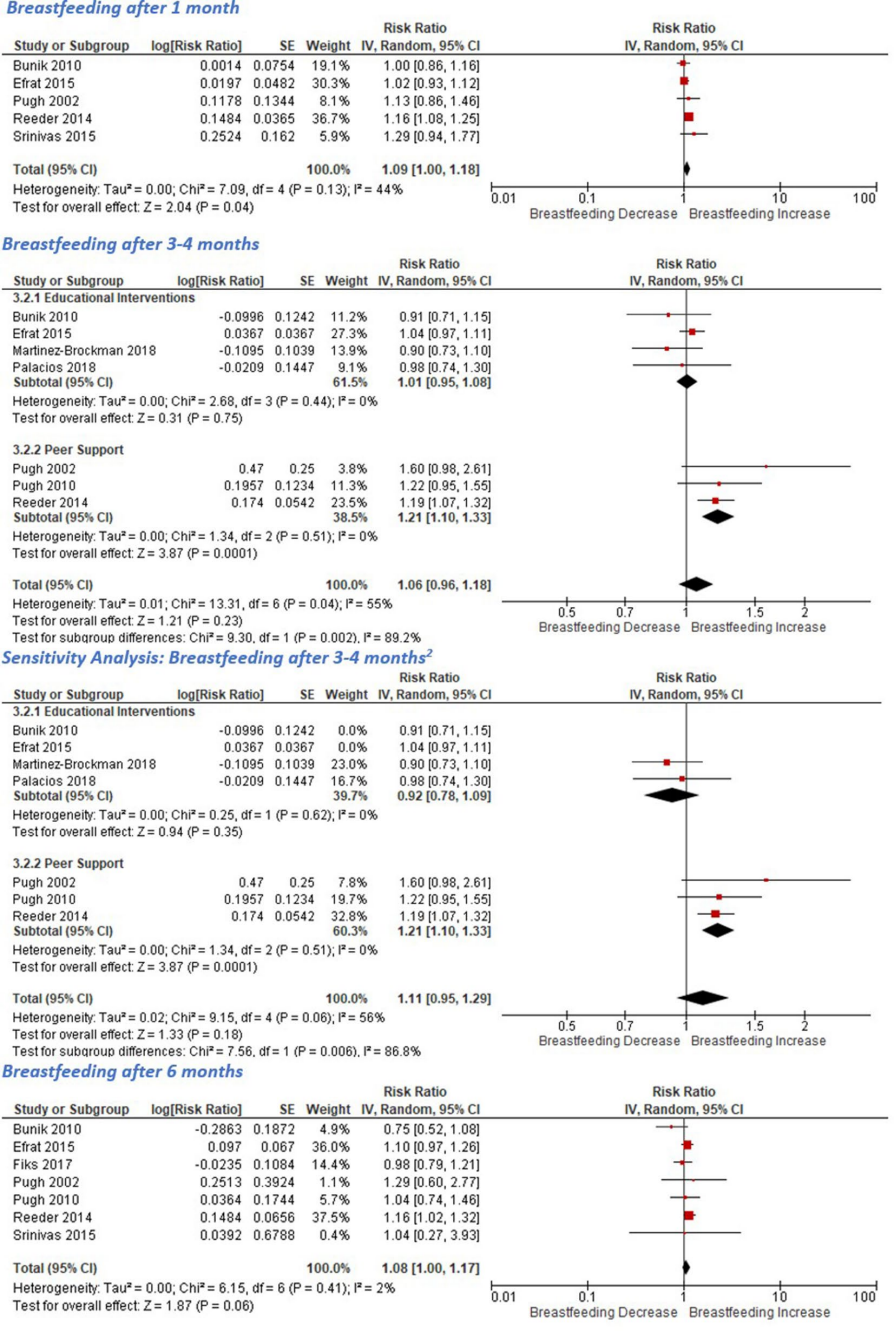
Effect of teleinterventions on breastfeeding

Five studies (all providing supportive phone calls) reported breastfeeding at one month [27, 31, 33, 34, 38]. Pooled results indicate these significantly increased breastfeeding (Fig. 4). A sensitivity analysis including only the three studies without a high risk of attrition bias enhanced this effect (RR 1.16, 95% CI [1.09,1.24], *P* < 0.0001) with minimal heterogeneity (I2 = 0%, *P* = 0.8).

Seven studies reported breastfeeding between 3 and 4 months [27, 31, 34, 35, 37–39]. Of these, one assessed passive educational text messages [37], one two-way motivational texts [35], two evaluated nurse phone calls [34, 38], and three provided telephone peer support [27, 31, 39]. On average, they did not increase breastfeeding at 3–4 months postpartum (Fig. 4). Heterogeneity was high and therefore the studies were divided into subgroups based on the main intervention component (education or peer support)(Fig. 4) [27, 31, 39].

Four studies utilised educational teleinterventions [34, 35, 37, 38]. These included uni- (where the mother could not respond) and bi-directional (where responses from the mother were answered) text messages [35, 37] and phone calls from lactation educators [38] or nurses [34]. One study provided just 2 weeks of postpartum nurse calls [34] whilst the other three were started antenatally and continued for > 4 m [35, 37, 38]. On average, these did not increase breastfeeding at 3–4 months (RR 1.01, 95% CI [0.95,1.08]), although high attrition (> 15%) in three contributing studies limits confidence in this finding [34, 35, 38]. Three papers assessed peer support teleinterventions. All evaluated phone calls for 4 + months postpartum [27, 31, 39], with only one implemented antenatally [31]. On average they significantly increased breastfeeding at 3–4 months (RR 1.21, 95% CI [1.1,1.33]). Results were homogenous (I^2^ = 0%, *P* = 0.51).

### 6 months

Only 7 studies had a 6 month follow up (far shorter than the WHO recommended breastfeeding duration of two years) [27, 31–34, 38, 39, 41]. Four were delivered postnatally [27, 33, 34, 39] and intervention duration ranged from 2 weeks [34] to 9 months [32]. Six studies provided phone calls [27, 31, 33, 34, 38, 39], whilst Fiks et al. created a multi-component Facebook peer group [32]. Pooled results indicate a modest improvement in breastfeeding at 6 months (Fig. 4), which was strengthened in a sensitivity analysis for attrition bias (RR = 1.10, 95% CI [1.00,1.22]).

### Quality assessment

Overall, the evidence quality was ‘very low’, with only ‘EBF at 1 month’ deemed ‘low’ quality. Evidence was rated down for high attrition bias without exploratory or compensatory analysis, and for insufficient allocation sequence blinding. Breastfeeding was self-assessed in all studies and blinding of the data collector was variable, introducing concerns of measurement bias. As it was unfeasible for most interventions, no outcome was downgraded for not blinding participants [41–43]. ‘Any breastfeeding at 3–4 months’ had high heterogeneity. Although subgroup analysis minimised this, divisions into subgroups may lead to misleading conclusions, so this outcome was downgraded for inconsistency [44]. Additionally, all outcomes were downgraded by one quality category for ‘imprecision’ due to insufficient power (recruited sample size below estimated) or lack of power analysis, with confidence intervals crossing the point of no difference (See Additional File Fig. 1).

## Discussion

This meta-analysis assessed the effect of teleinterventions on breastfeeding in LIW in HIC. Nine studies were included, the majority of which used mobile phones to deliver educational or peer support (Table 1). Intervention success was variable and implementation times ranged from 2 weeks to 9 months [32, 34]. Our results indicate teleinterventions modestly increase EBF at 3-4months postpartum and any breastfeeding at 1 and 6 months postpartum while a particular intervention, peer support in contrast with educational interventions, showed the strongest effect at 3–4 months postpartum. All the eligible studies were conducted in the US and most were of poor quality.

It is known that teleinterventions improve breastfeeding in the wider maternal population, but this is the first systematic review and meta-analysis of their effect on breastfeeding in LIW, who are neglected in the literature [6, 8]. Six studies measured EBF [27, 31–33, 37, 38], but only five had a follow-up period lasting for the WHO recommended 6 months [27, 31–33, 38, 45]. Promisingly, their pooled results mirrors research in the wider maternal population, suggesting that teleinterventions may increase EBF [6, 8]. Interestingly, our analysis indicated that teleinterventions were not as effective for LIW as in the general population. A meta-analysis of teleinterventions for all mothers identified a three-fold increase in EBF at 6 months (*P* = 0.001) [6], whereas our analysis indicated only a minimal positive effect. It is not clear whether the weaker effect in LIW results from the low quality of studies or if it reflects a true lower potential for teleinterventions in this subgroup. The latter might suggest more intense interventions might be needed to promote breastfeeding in LIW, and there is an urgent need for more methodologically sound RCTs to explore this.

Despite prior reviews indicating longer interventions durations are more effective, only four teleinterventions were implemented for six months [8, 27, 32, 38, 39]. Those that were implemented for six months or longer doubled EBF [27, 32, 38], demonstrating LIW may also benefit greatly from sustained remote support.

Interestingly, although in general teleinterventions did not show evidence of effect on ‘any breastfeeding’ at 3 months, there was a stark difference between studies providing educational or peer support. This meta-analysis included four educational teleinterventions delivered by either nurses [34] or ‘specifically trained lactation educators’ [38]. Only one of these increased breastfeeding and high study attrition (42.5% retention at 6 months) severely limits their result’s reliability [38]. Unsurprisingly, our pooled average indicates that these educational teleinterventions do not increase breastfeeding in LIW at 3–4 months.

This reflects the results of a study in more affluent mothers which established that educational support had little effect on breastfeeding beyond 2 months postpartum [19]. Likewise, a Cochrane review also found that additional antenatal education did not significantly increase breastfeeding duration [41]. This is perhaps unsurprising, as educational interventions are founded on the assumption that mothers will choose to breastfeed for longer if they have a better understanding of breastfeeding’s benefits [41]. However, interviews with LIW indicate they are already aware of these and, rather than lack of information, low rates reflect wider socio-structural constraints that remain unaddressed by educational interventions [13, 15, 46].

Poor study designs may also contribute to the apparent inefficacy of the educational teleinterventions in this review. Text messages in the Martinez-Brockman et al. and Palacious et al. studies were pre-scripted, as were phone calls provided by Bunik et al., which were also regularly audited to ensure they followed protocol [34, 35, 37]. This improves fidelity but limits personalisation, so the advice given may have been irrelevant and unhelpful. This design is interesting and potentially self-limiting, as the literature strongly favours personalisation. A review of breastfeeding support for all women identified that flexible telephone interventions better promoted breastfeeding compared to those with a standardised format, and our results strengthen this argument [47].

There was large variation in timing, nature, and implementation fidelity between studies providing peer support at 3–4 months. However, our subgroup analysis at 3–4 months suggests remote peer support can more effectively increase breastfeeding in LIW than traditional interventions. Interestingly, although neither Srinivas et al. and Reeder et al. reached the number of calls specified in their protocols, their low-intensity interventions increased breastfeeding [31, 33]. Support networks are important for LIW but are often unavailable [42, 48]. It appears continuous remote contact with a role model, however infrequent, may provide these, empowering mothers to overcome structural barriers thereby increasing breastfeeding [16, 49]. The added flexibility of teleinterventions may have also allowed the mother to access help when they needed it, rather than at prescribed timepoints.

Although the efficacy of peer support for increasing breastfeeding is well established, it is encouraging that they appear as efficacious when delivered remotely. Only Fiks et al. combined group support with education from medical personnel [32]. Their online Facebook group created a virtual environment that normalised breastfeeding, which itself is strongly associated with a longer breastfeeding duration [32, 42, 50]. Interestingly, their study was the only complex teleintervention for LIW in HIC. This is concerning given multi-component interventions are known to be more effective at promoting breastfeeding, and may improve teleintervention’s efficacy in a population with a particularly high risk of early discontinuation [42].

## Limitations

Despite the expansive potential of modern technology, most interventions used telephone calls or texts [27, 31, 33–35, 37–39, 51]. Increasingly healthcare teleinterventions utilise multiple technologies, which may be particularly useful for breastfeeding (as suggested by Fiks et al.’s positive findings) [32, 52]. Focus on telephone calls and texts in the literature limits the generalisability of this review to these relatively simple delivery modalities.

All studies were published in the US, so results may only be applicable to low-income Americans. Most study participants were ethnic minorities (disproportionately Hispanic women [5/9 studies]) which may reflect the reality that LIW in HIC are often also ethnic minorities [53]. However, as susceptibility to breastfeeding interventions varies between ethnicities, these population demographics also limit generalisability of our findings [53, 54]. Certain ethnicities are overrepresented in the literature and more breastfeeding research with diverse participants is sorely needed. Overrepresentation of certain ethnicities reflects the wider breastfeeding literature, and there is a need to increase the diversity of minority representation in breastfeeding research in HIC [53].

The dearth and low quality of eligible studies limited this review’s reliability and power and prevented further exploration of the pooled-results (such as the potential effect modification of ethnicity or intervention route) and meta-regression. Our search strategy was comprehensive so the limited number of studies reflects the paucity of breastfeeding research for LIW [55]. This reinforces previous findings, indicating they are sorely neglected in current research [5, 53].

Breastfeeding definitions in the eligible studies were heterogeneous, a recurring problem in the breastfeeding literature [56]. ‘Usual’ care in the control group was also inconsistent and poorly defined across all studies, and both limit interstudy comparability. Varying control care may contribute to the high heterogeneity in some of our pooled averages. More intensive care can lead to higher background breastfeeding in the control group and so successful teleinterventions would have a proportionately smaller impact and require a larger study population to detect it. However, most studies suffered from a small sample size and high attrition– yet they did not employ suitable compensatory designs or analysis [27, 33–35, 38]. Accordingly, most were underpowered to detect changes in breastfeeding (Additional File Table 4).

Inclusion of these underpowered studies might explain the overall lack of statistical significance of our the results, which contrast with the significant positive findings from previous reviews in the wider maternal population [6]. This is likely, given that the pooled averages at all time points increased in a sensitivity analysis which removed studies with the highest bias and lowest power (although it did not change their statistical significance). Therefore, as our pooled averages are a conservative estimate, it is likely teleinterventions can improve breastfeeding in LIW, more effectively than usual care.

## Conclusion

This meta-analysis shows that teleinterventions can increase any and exclusive breastfeeding in LIW up to 6 months postpartum. This is encouraging, as even small increases in breastfeeding are associated with significant health benefits for both mothers and their children. Further confirmatory research in other HIC with higher methodological quality, longer follow-up durations (at least six months), and more ethnic diversity will help define how teleinterventions can best fulfil their potential to support and empower more LIW to breastfeed.

### Electronic supplementary material

Below is the link to the electronic supplementary material.

Supplementary Material 1

## Data Availability

The datasets used and/or analysed during the current study are available from the corresponding author on reasonable request.

## Abbreviations

CI: Confidence Intervals
EBF: Exclusive Breastfeeding
GRADE: Grading and Recommendations Assessment, Development, and Evaluation
HIC: High-income countries
LIW: Low-income women
MeSH: Medical Searching Heading
PRISMA: Preferred Reporting Items for Systematic Reviews and Meta-Analyses
RoB2: Revised Cochrane risk-of-bias tool for randomised trials
RR: Risk Ratios
UK: United Kingdom
US: United States
WHO: World Health Organisation
