# Supplementary material for: Teleintervention’s effects on breastfeeding in low-income women in high income countries: a systematic review and meta-analysis
Source: Int Breastfeed J. 2024 Apr 13;19:26. doi: 10.1186/s13006-024-00631-2 (PMC11015560; doi:10.1186/s13006-024-00631-2)
Supplement: Supplementary file 1 — Supplementary Material 1 [file 13006_2024_631_MOESM1_ESM.docx]

Additional Files

Pubmed Final Search Strategy .txt

(((((breastfeed* OR lactat* OR "breast feed*" OR breast-feed* OR breastfeeding OR Lactation) AND (promote OR promotion OR initiation OR initiate OR support OR sustain)) AND (low-income OR "low income" OR "depri*" OR "low SES" OR 'poverty' OR "low socio-economic status" OR "lowest quartile" OR "low-income" OR "lowest decile" OR "low maternal income" OR "low socioeconomic status" OR "low education"))) AND (online OR remote OR tele* OR web-based OR technolog* OR website OR video OR app OR "web based" OR zoom OR text OR mobile OR phone OR "social media*" OR "online support*" OR e-health OR computer OR e-technology OR facebook OR multimedia OR multi-media OR Telemedicine)) AND (randomizedcontrolledtrial[Filter])

Additional File Table 1 - PICO and MeSH Search Terms. txt

|  | **Elements** | **Keyword** | **Search Terms** | **MeSH Terms** | **MeSH**  **Headings** |
| --- | --- | --- | --- | --- | --- |
| **P** | Low-income mothers  Low education | Low income  Low SES  Deprived  Poverty  Low education | low-income  low income  depri*  low SES  poverty  low socio-economic status  lowest quartile  low-income  lowest decile  low maternal income  poor  low socioeconomic status | Low Income Population  Low-Income Population | Poverty    Maternal Deprivation    Paternal Deprivation |
| **I** | Online/Technology based breastfeeding promotion | Online  Tele-  Technology  Phone | \| Online  remote  tele*  web-based  technolog*  website  telephone \| video  app  web based  zoom  text  mobile  phone  social media* \| online support*  e-health  computer  e-technology  facebook  multimedia  multi-media  combined \| \| --- \| --- \| --- \| | Mobile Health  Telehealth  eHealth  mHealth    Social Medium  Twitter Messaging  Web 2.0    Short Message Service  Text Messages  Texting | Telemedicine    Social Media    Text Messaging |
| **C** | No intervention/ Control |  |  |  |  |
| **O** | Improved breastfeeding rates | Breastfeeding  Sustain  Promote | \| breastfeed*  lactat*  breast feed*  breast-feed* \| AND \| promote  Promotion  Initiation  initiate  Support  sustain \| \| --- \| --- \| --- \| | Breast Fed  Breast Feeding, Exclusive  Breastfed  Breastfeeding  Breastfeeding, Exclusive  Milk Sharing  Wet Nursing | Breast Feeding |

Additional File Table 2 – Rational for Study Exclusion after Full Text Analysis .txt

| **Reference** | **Title** | **Reason for Exclusion** |
| --- | --- | --- |
| Arch Pediatr Adolesc Med. 2005 Sep;159(9):836-41. doi: 10.1001/archpedi.159.9.836. | A randomized trial assessing the efficacy of peer counselling on exclusive breastfeeding in a predominantly Latina low-income community. | Intervention does not meet inclusion criteria - Mostly delivered in person |
| Kellams AL, Gurka KK, Hornsby PP, Drake E, Conaway MR. A Randomized Trial of Prenatal Video Education to Improve Breastfeeding Among Low-Income Women. Breastfeed Med. 2018 Dec;13(10):666-673. doi: 10.1089/bfm.2018.0115. Epub 2018 Oct 23. PMID: 30351169. | A Randomized Trial of Prenatal Video Education to Improve Breastfeeding Among Low-Income Women | Intervention does not meet inclusion criteria - Video shown in clinic |
| Birth. 2002 Jun;29(2):95-100. doi: 10.1046/j.1523-536x.2002.00169.x. | Breastfeeding duration, costs, and benefits of a support program for low-income breastfeeding women | Duplicate |
| BMC Pregnancy Childbirth. 2016 Jun 4;16(1):136. doi: 10.1186/s12884-016-0914-z. | Can a community health worker administered postnatal checklist increase health-seeking behaviors and knowledge?: evidence from a randomized trial with a private maternity facility in Kiambu County, Kenya | Not conducted in high income country |
| Cell phone based peer counselling can support exclusive breastfeeding: A  randomized controlled trial in Kenya Sellen D.; Mbugua S.; Webb-Girard A.; Lou W.; Duan W.; Kamau-Mbuthia E. FASEB Journal (2014) 28:1 SUPPL. 1. Date of Publication: April 2014 | Cell phone based peer counselling can support exclusive breastfeeding: a randomized controlled trial in Kenya | Not conducted in high income country |
| Health Educ Res. 2002 Oct;17(5):531-40. doi: 10.1093/her/17.5.531. | Design of an intervention addressing multiple levels of influence on dietary and activity patterns of low-income, postpartum women. | No results |
| Gross SM, Caulfield LE, Bentley ME, Bronner Y, Kessler L, Jensen J, Paige VM. Counseling and motivational videotapes increase duration of breast-feeding in African-American WIC participants who initiate breast-feeding. J Am Diet Assoc. 1998 Feb;98(2):143-8. doi: 10.1016/s0002-8223(98)00037-6. PMID: 12515413. | Counseling and motivational videotapes increase duration of breast-feeding in African-American WIC participants who initiate breast-feeding | Intervention does not meet inclusion criteria - Intervention delivered in a clinic |
| Lewkowitz AK, López JD, Werner EF, Ranney ML, Macones GA, Rouse DJ, Savitz DA, Cahill AG. Effect of a Novel Smartphone Application on Breastfeeding Rates Among Low-Income, First-Time Mothers Intending to Exclusively Breastfeed: Secondary Analysis of a Randomized Controlled Trial. Breastfeed Med. 2021 Jan;16(1):59-67. doi: 10.1089/bfm.2020.0240. Epub 2020 Oct 20. PMID: 33085510; PMCID: PMC7826429. | Effect of a Novel Smartphone Application on Breastfeeding Rates Among Low-Income, First-Time Mothers Intending to Exclusively Breastfeed: Secondary Analysis of a Randomized Controlled Trial | Subgroup analysis of separate study |
| BMC Pediatr. 2018 Oct 30;18(1):337. doi: 10.1186/s12887-018-1308-3. | Early contact versus separation: effects on mother-infant interaction one year later | Study did not target breastfeeding |
| Matern Child Nutr. 2018 Jan;14(1):e12488. doi: 10.1111/mcn.12488. Epub 2017 Aug 1. | Feasibility and acceptability of a text message intervention used as an adjunct tool by WIC breastfeeding peer counsellors: The LATCH pilot | Pilot Study of included paper |
| Arch Pediatr Adolesc Med. 2004 Sep;158(9):897-902. doi: 10.1001/archpedi.158.9.897. | Effectiveness of breastfeeding peer counselling in a low-income, predominantly Latina population: a randomized controlled trial | Intervention does not meet inclusion criteria - mostly delivered in person |
| Uscher-Pines L, Ghosh-Dastidar B, Bogen DL, Ray KN, Demirci JR, Mehrotra A, Kapinos KA. Feasibility and Effectiveness of Telelactation Among Rural Breastfeeding Women. Acad Pediatr. 2020 Jul;20(5):652-659. doi: 10.1016/j.acap.2019.10.008. Epub 2019 Oct 16. PMID: 31629118. | Feasibility and Effectiveness of Telelactation Among Rural Breastfeeding Women | Study population not low income |
| Kim SS, Nguyen PH, Tran LM, Sanghvi T, Mahmud Z, Haque MR, Afsana K, Frongillo EA, Ruel MT, Menon P. Large-Scale Social and Behaviour Change Communication Interventions Have Sustained Impacts on Infant and Young Child Feeding Knowledge and Practices: Results of a 2-Year Follow-Up Study in Bangladesh. J Nutr. 2018 Oct 1;148(10):1605-1614. doi: 10.1093/jn/nxy147. PMID: 30169665; PMCID: PMC6168701. | Large-Scale Social and Behavior Change Communication Interventions Have Sustained Impacts on Infant and Young Child Feeding Knowledge and Practices: Results of a 2-Year Follow-Up Study in Bangladesh | Intervention does not meet inclusion criteria – Assessed intensity of Alive and Thrive programme (a complex multicomponent intervention with in-person components). |
| PLoS Med. 2019 Oct 24;16(10):e1002939. doi: 10.1371/journal.pmed.1002939. eCollection 2019 Oct. | mHealth intervention "ImTeCHO" to improve delivery of maternal, neonatal, and child care services-A cluster-randomized trial in tribal areas of Gujarat, India | Study population not low income |
| Forster DA, McLardie-Hore FE, McLachlan HL, Davey MA, Grimes HA, Dennis CL, Mortensen K, Moorhead AM, Tawia S, Gold L, Shafiei T, Small R, East CE, Amir LH. Proactive Peer (Mother-to-Mother) Breastfeeding Support by Telephone (Ringing up About Breastfeeding Early [RUBY]): A Multicentre, Unblinded, Randomised Controlled Trial. EClinicalMedicine. 2019 Mar 6;8:20-28. doi: 10.1016/j.eclinm.2019.02.003. PMID: 31193656; PMCID: PMC6537529. | Proactive Peer (Mother-to-Mother) Breastfeeding Support by Telephone (Ringing up About Breastfeeding Early [RUBY]): A Multicentre, Unblinded, Randomised Controlled Trial | Study population not low income |
| Pediatrics. 2005 Dec;116(6):1413-26. doi: 10.1542/peds.2005-0435. | Randomized, controlled trial of a prenatal and postnatal lactation consultant intervention on duration and intensity of breastfeeding up to 12 months | Intervention does not meet inclusion criteria - mostly delivered in person |
| Pediatr Obes. 2019 Jan;14(1):10.1111/ijpo.12456. doi: 10.1111/ijpo.12456. Epub 2018 Sep 17. | 'Ripple' effect on infant zBMI trajectory of an internet-based weight loss program for low-income postpartum women | Study did not target breastfeeding rates |
| BJOG. 2018 Nov;125(12):1620-1629. doi: 10.1111/1471-0528.15337. Epub 2018 Aug 28. | Short message service communication improves exclusive breastfeeding and early postpartum contraception in a low- to middle-income country setting: a randomised trial | Study population not low income |
| Shaw E, Kaczorowski J. The effect of a peer counselling program on breastfeeding initiation and longevity in a low-income rural population. J Hum Lact. 1999 Mar;15(1):19-25. doi: 10.1177/089033449901500108. PMID: 10578771. | The effect of a peer counseling support program on breastfeeding initiation, duration and exclusivity among low-income Hispanic women. | Study dissertation, not RCT |
| Am J Dis Child. 1990 Apr;144(4):471-4. doi: 10.1001/archpedi.1990.02150280093019. | The effect of postpartum lactation counselling on the duration of breast-feeding in low-income women. | Intervention does not meet inclusion criteria – Published before 2000 and full text unavailable |
| Pan Afr Med J. 2015 Jun 22;21:138. doi: 10.11604/pamj.2015.21.138.7026. eCollection 2015. | The effect of prenatal counselling on postpartum family planning use among early postpartum women in Masindi and Kiryandongo districts, Uganda | Study did not target breastfeeding rates |
| Lancet Glob Health. 2019 Mar;7(3):e357-e365. doi: 10.1016/S2214-109X(18)30494-7. | The effect of the Alive & Thrive initiative on exclusive breastfeeding in rural Burkina Faso: a repeated cross-sectional cluster randomised controlled trial | Intervention does not meet inclusion criteria – Intervention mostly delivered in person. |
| Lewkowitz AK, López JD, Carter EB, Duckham H, Strickland T, Macones GA, Cahill AG. Impact of a novel smartphone application on low-income, first-time mothers' breastfeeding rates: a randomized controlled trial. Am J Obstet Gynecol MFM. 2020 Aug;2(3):100143. doi: 10.1016/j.ajogmf.2020.100143. Epub 2020 May 17. PMID: 33345878. | Impact of a novel smartphone application on low-income, first-time mothers’ breastfeeding rates: a randomized controlled trial | Control group also given breastfeeding teleintervention (not usual care). |

Additional File Table 3 - RoB 2 Bias Domains .txt

| **RoB 2 Bias Domains** |
| --- |
| - Randomisation - Deviation from the intended intervention - Missing outcome data - Outcome measurement - Selection of results reported |

Additional File TABLE 4 - Full Rob 2 Risk of Bias Rating Rationale .txt

| **Study** | **D1** | **D2** | **D3** | **D4** | **D5** |
| --- | --- | --- | --- | --- | --- |
| Bunik | - Random block allocation - Allocated through 'sequentially numbered opaque sealed envelopes' (acceptable process)   - Use of opaque envelopes and sequential opening minimises risk of manipulation - No information on the sequence generation   - Increased selection bias risk - Similar recorded demographics between groups   - Recorded parity and delivery mode | - Blinding of participants and researchers unfeasible as the intervention was telephone calls   - High bias risk - Phone calls standardised in intervention group using script. - Usual care for control group not described.   - Difficult to determine likelihood of additional non-protocol interventions. - Stated analysis is ‘Intention-to-treat analysis’ however excluded loss to follow up and dropouts   - Thus conducted a ‘per-protocol’ analysis   - Increasing attrition bias risk - 6 months attrition rate >20% severely compromising validity of results - No information on missing data analysis/exploration | - Attrition <20% at 1m but >20% at 6m (30%) with no compensatory analysis for missing data - Low confidence in results validity - No sensitivity analysis to determine if LTFU characteristics significantly differed from retained participants. | - Self-reported breastfeeding data - subject to recall and performance bias - There is no information on who conducted the interview   - Risk of enhanced performance bias in the intervention group if it was collected by the peer councillor   - The mother may have built a rapport and therefore be more willing to please and over-reporting breastfeeding than a mother in the control group. - High risk of Hawthorne effect as benefits of breastfeeding were included in the intervention   - Mothers in the intervention group may be disproportionately likely to over-report breastfeeding - No information of the questionnaire used to asses feeding status – increased risk of reporting bias and unconscious leading of the participant by the researcher | - Pre-study analysis protocol unavailable   - Only available analysis information was published alongside results - unable to determine if analysis followed pre-specified protocol - Many analysis methods are stated in the paper methods is little information on their results – risk of selective reporting   - Unlikely given the paper concluded the intervention had no effect on breastfeeding. |
| Efrat | - Computer software determined group allocation through block randomisation (best practice)   - No information on the computer software so cannot confirm if allocation completely random. - Baseline and demographic characteristics had no statistically significant differences in any characteristics between groups (P>0.05) | - Blinding of participants and researchers unfeasible as the intervention was telephone calls   - High bias risk - Low intervention fidelity despite regular evaluation   - Phone calls over- and under-delivered so hard to gauge impact of deviation on results - Low risk of additional non-protocol interventions   - Selective recruitment from areas without an existing peer supports service - Per-protocol analysis excluding disenrolled mothers and those lost to follow up | - High attrition - Data only available for 38.5% and 42.4% of original control and intervention group respectively. - Participant loss explored (fishers test and sensitivity analysis) but results not presented - However, mothers who stopped breastfeeding were disenrolled, significantly positively biasing their results | - Self-reported breastfeeding data - subject to recall and performance bias - Data collector unblinded research assistant - Each research assistant was assigned a control and intervention participant, standardising data collection through a pre-determined questionnaire   - No information on script   - Introduces opportunity for researchers to unconsciously ask more leading questions in the intervention than the control group and thus might have influenced data collection (leading to reporting bias). - Risk of reporting bias due to lack of blinding mitigated through identical data collection strategies between groups.   - Slight potential of unintentional guiding of participants - Data collectors provided with training to minimise bias   - But acknowledged that the rapport established between the intervention group and the research assistants (who also delivered the intervention) is likely to have led to over-reporting of breastfeeding rates in the intervention group - performance and recall bias | - Prior analysis intentions unavailable - Acknowledged protocol deviation and creation of a separate subcategory mid-trial ('Currently Exclusively Breastfeeding')   - Response guided – pragmatic but introduces bias in favour of the intervention   - However it may decrease mismeasurement bias (as initial formula feeding in hospital not decided by mothers, who exclusively breastfed at home) - Risk of selective reporting as data on breastfeeding duration collected but not presented |
| Fiks | - Block randomisation conducted with varying block sizes - Statistician externally generated allocation sequence (random and computer generated)   - Best practice - Allocation through sealed security envelopes, although no information provided on the order in which they were opened. - Neither staff nor participants were aware of treatment allocation before study - Similar baseline characteristics with roughly the same recruitment ratio as pre-specified in the study power analysis. | - Blinding of participants and researchers unfeasible   - Increases risk of bias - Per-protocol analysis but high retention rate so missing data unlikely to impact results - No information regarding other treatments participants may have sought - Additional non-protocol interventions unlikely as intervention engagement was high, intensive, and implemented following the protocol | - High retention rate - Data available for 85/87 participants randomised (97.7%) - Likely no bias from missing data | - Self-reported breastfeeding data - subject to recall and performance bias - Standardised and validated questionnaire - Infant Feeding Style Questionnaire (IFSQ)   - Validated for assessing maternal beliefs and feeding behaviours in an African American population (18) - Online survey reduces risk of unconscious researchers bias and performance bias in the intervention group, as they may feel less pressure to report the 'ideal outcome' (breastfeeding) than if data collected verbally (76). | - Only available analysis information was published alongside results - unable to determine if analysis followed pre-specified protocol - Study was extended an addition 3 months beyond protocols specified time.   - However, no results were reviewed before this decision so unlikely to have influenced result analysis or introduced bias - Protocol suggests a separate breastfeeding survey should have been used   - This is not described in the paper   - Difficult to determine if separate survey used and unreported (selective reporting bias) or if they deviated from the protocol when collecting data (and did not administer the survey) |
| Martinez-Brockman | - No information on randomisation method or allocation provided - Participants actively recruited but by their own peer counsellor.   - Potentially increases selection bias as the peer counsellor may have encouraged certain women with particular characteristics more than others.   - Residual confounders may therefore be unequally distributed between groups and result generalisability is limited. - Slight unexplained difference in group sizes (intervention = 94, control = 80)   - Potentially indicates selection bias - Baseline demographic characteristics were similar between both study arms with no statistically significant differences between groups (p>0.05 for all characteristics) | - Blinding of participants and researchers unfeasible   - Increases risk of bias - High intervention fidelity as text message pre-scheduled and content pre-screened to ensure matched protocol - No information regarding additional interventions delivered/sought - ‘As-treated analysis’ used with high attrition rate (39.2%)   - Compensatory regression analysis increases confidence in findings and reduces attrition bias | - Data at 3 months only available for 60.8% of randomised participants   - Over the 20% threshold proposed by Schultz as seriously compromising result's validity (102) - Missing data adjusted for using multivariate ordinal and binary longitudinal modelling, sensitivity analyses and regression analyses - Characteristics associated with non-differential results controlled for with regression analysis   - Decreases impact of attrition bias on results (57,94) | - Standardised and validated questionnaire - Infant Feeding Practices Study II survey   - Decreases measurement bias - Response bias minimised by standardised script delivered by blinded interviews   - Decreases risk of response bias   - However, mothers receiving the intervention may be more aware of the benefits of breastfeeding and therefore feel increased pressured to over-report their breastfeeding (performance bias) | - Study protocol specified a follow up time of 6 months for changes in exclusive breastfeeding   - Only 3 month follow up reported, deviating from the protocol – indicates selective reporting bias - Although data for 6 months is unavailable, breastfeeding status at 2 weeks and 3 months were reported as intended. - No information on intended analysis in the protocol. It is extensively described in the methods of the published paper, and presented results are concordant with this. |
| Palacious | - Computer generated random number sequence   - Participants sequentially assigned ID number as enrolled   - Random computer-generated sequence was then matched IDs with blocks of 2-6 participants (random block allocation)     - This effectively blinded the allocation sequence. - Baseline demographic characteristics were similar between intervention and control groups with no statistically significant differences in any of the recorded characteristics. | - Both the intervention and control groups received text messages. Control group messages covered general infant health (not infant feeding) and therefore it is plausible that participants in both groups were blinded.   - Breastfeeding secondary outcome – may have reduced performance bias - Text messages were produced by the United States Department of Agriculture’s Food and Nutrition Service and sent automatically with no knowledge of the recipients. - Per-protocol analysis - Inappropriate as it increases risk of attrition bias.   - Somewhat mitigated by including site as covariate to reduce residual confounding. - 30% of participants (n = 32) were not analysed at 4 months. This is over the 20% threshold which poses a 'serious threat to validity (102) | - High attrition rate - Data for 30% unavailable at 4 months (32/202 initially randomised)   - Seriously compromises results validity (102) - Reasons for participant loss explored but not controlled for in analysis   - May positively bias results as white mothers more likely retained and also more likely to breastfeed - Similar attrition between control and intervention group maintains confounding balance achieved during randomisation   - Mitigates impact of confounding biases | - Self-reported breastfeeding data - subject to recall and performance bias - Standardised, robust, validated questionnaire administered by blinded data collector (103) - Infant food frequency questionnaire (infant FFQ)   - Decreases risk of performance and reporting bias in the intervention group   - Infant FFQ does not exclusively focus on breastfeeding, reducing risk of performance bias as the 'ideal outcome' (breastfeeding) may have been less obvious to participants. - Intervention group text messages contained information on the benefits of breastfeeding, however it also covered other aspects of healthy infant behaviours   - Reduces risk of performance bias in intervention group as no obvious ideal answer | - Deviation from protocol suggest selective reporting   - Protocol indicated separate breastfeeding duration questionnaire but results not published   - Protocol outlined a 6-month timespan, however trial only reported to 4 months - Study meets all analysis intentions stated in the final paper but cannot ensure these were the same before trial completion |
| Reeder | - "Participants were allocation through a computer-generated random number function   - No further information is provided on how this sequence was implemented however, raising concerns of selection bias. - Recorded baseline characteristics similar between control and intervention groups, increasing confidence in randomisation process | - Peer support is an active two-way intervention – blinding is unfeasible   - May positively skew results. - The supplementary information indicated that data on intervention protocol adherence was collected and not analysed - Indicates potential protocol deviation. - Concern mitigated by disclosure of other protocol deviations (most mothers were not contacted with a week of delivery). - Lack of peer-support groups in surrounding geographical area reduced probability of non-protocol additional treatment) - Per-protocol analysis but high retention rate so missing data unlikely to impact results - Both intervention arms were analysed together   - Decision made after initial analysis of results – increasing bias risk | - Likely no bias from missing data as 96.8% of randomised participants retained and analysed | - Self-reported breastfeeding data - subject to recall and performance bias - Breastfeeding information collected in a standardised format by an external blinded data collector body, blinding collection   - Minimising researcher bias - Intervention group mothers received more information on the benefits of breastfeeding – increasing performance bias   - Differential impact hard to gauge as breastfeeding is promoted during usual care, so all groups were likely equally aware of the advantages. | - Both intervention arms were analysed together however unlikely to significantly impact study conclusions on overall intervention efficacy - Study meets all analysis intentions stated in the final paper but cannot ensure these were the same before trial completion |
| Pugh 2002 | - Allocation through sealed envelope technique - No information on order of opening or generation of allocation sequence - Recorded baseline characteristics similar between both arms | - Peer support is an active two-way intervention – blinding is unfeasible - May positively skew results. - No deviations described. - Unlikely non-protocol interventions were received, given that the control group received the usual standard of care. - Per-protocol analysis used but high retention (40/41 participants) limits bias risk | - No information on number of participants included in the analysis   - Presented results indicated one participant was lost to follow up and excluded from analysis (recruited participants n = 41, percentages quoted indicate analysis n = 40). - No information on additional analysis to compensate for missing data provided - However low attrition decreases risk of bias from missing data | - Self-reported breastfeeding data - subject to recall and performance bias - No information on the data collector or the questions/survey – omission of this data indicates potential bias in favour of the intervention | - No statistical analysis for breastfeeding described (As it was a secondary outcome)   - Raises serious concerns regarding data handling and selective reporting bias |
| Pugh 2010 | - Study statistics generated allocation sequence using randomised computer (SPSS) model   - Best practice as impossible to predict allocation - Sealed envelope technique (grouping into blocks of ten)   - Likely concealed allocation sequence   - No further information of envelope characteristics in sealed envelope technique (r.e opacity, opening order, tamper proof) - Allocation sequence at risk of compromise (104). - Baseline characteristics did not significantly differ between groups - Similar sample sizes were similar indicate effective randomisation | - Peer support is an active two-way intervention – blinding is unfeasible   - May positively skew results   - Increases risk of differential measurement bias - Contamination bias is unlikely as mothers were recruited from a wide area (25 miles of either hospital) and over an extended time period (Oct 2003 - Dec 2005). - No deviations are described.   - Unlikely non-protocol interventions were received, given that the control group received the usual standard of care. - Intention to treat analysis used with conservative reporting of those lost to follow up (reported as not breastfeeding) - minimising expectation bias. | - All participants analysed   - Assumed those lost to follow up discontinued breastfeeding – assumption may be erroneous and negatively skew results (73) | - Self-reported breastfeeding data - subject to recall and performance bias - No standardised survey and data collection by unblinded peer counsellor   - Very high risk of performance and detection bias due to pre-established rapport and potential for leading questioning (105) | - Of the pre-specified primary outcomes only breastfeeding duration is not reported   - Unlikely to bias overall conclusion as both proportion and odds of breastfeeding at the specified times were provided. - Unadjusted OR were not described however crude proportions were included so crude OR can be deduced and selective reporting is unlikely |
| Srinivas | - No information on the allocation sequence - Participants were randomised into blocks of four using block randomisation   - Small block sizes may have led to a predictable allocation sequence, increasing risk of selection bias given the small sample size (n = 130) and previous stratification based on breastfeeding intentions (57) - Additional risk of volunteer-bias as recruitment was passive, mothers not actively recruited but volunteered - Baseline characteristics did not significantly differ between groups increasing confidence in allocation and randomisation process | - Peer support is an active two-way intervention – blinding is unfeasible - May positively skew results. - Passive recruitment increases risk of additional non-protocol interventions in the control group as mothers may be more proactive and dissatisfied with their allocation to the control group - Resultant care may have increased breastfeeding in control group, decreasing study power and negatively skewing study results - Complete-case analysis increases bias as sample may no longer be representative of wider low-income population - However high retention rate (85% at 6 months) somewhat mitigates this risk as missing data unlikely to significantly impact results | - Potential attrition bias   - Data for 85.8% of randomised participants analysed   - Missing data not explored   - >5% participants lost - may significantly impact results (102) | - Self-reported breastfeeding data - subject to recall and performance bias - No standardised survey and data collection by unblinded peer counsellor in intervention group   - Very high risk of performance and detection bias due to pre-established rapport and potential for leading questioning (9) - Positive skewing of results likely as data collectors different between groups (peer counsellor in the intervention and research assistant for the control group)   - Intervention group has a higher risk of performance and reporting bias as they may have felt the need to appease their peer counsellor   - Control group is unlikely to have felt the same pressure and is at a lower risk of performance bias   - May inflate/generate breastfeeding difference between groups leading to inaccurate results and conclusions | - Only available analysis information was published alongside results - unable to determine if analysis followed pre-specified protocol |

Additional File
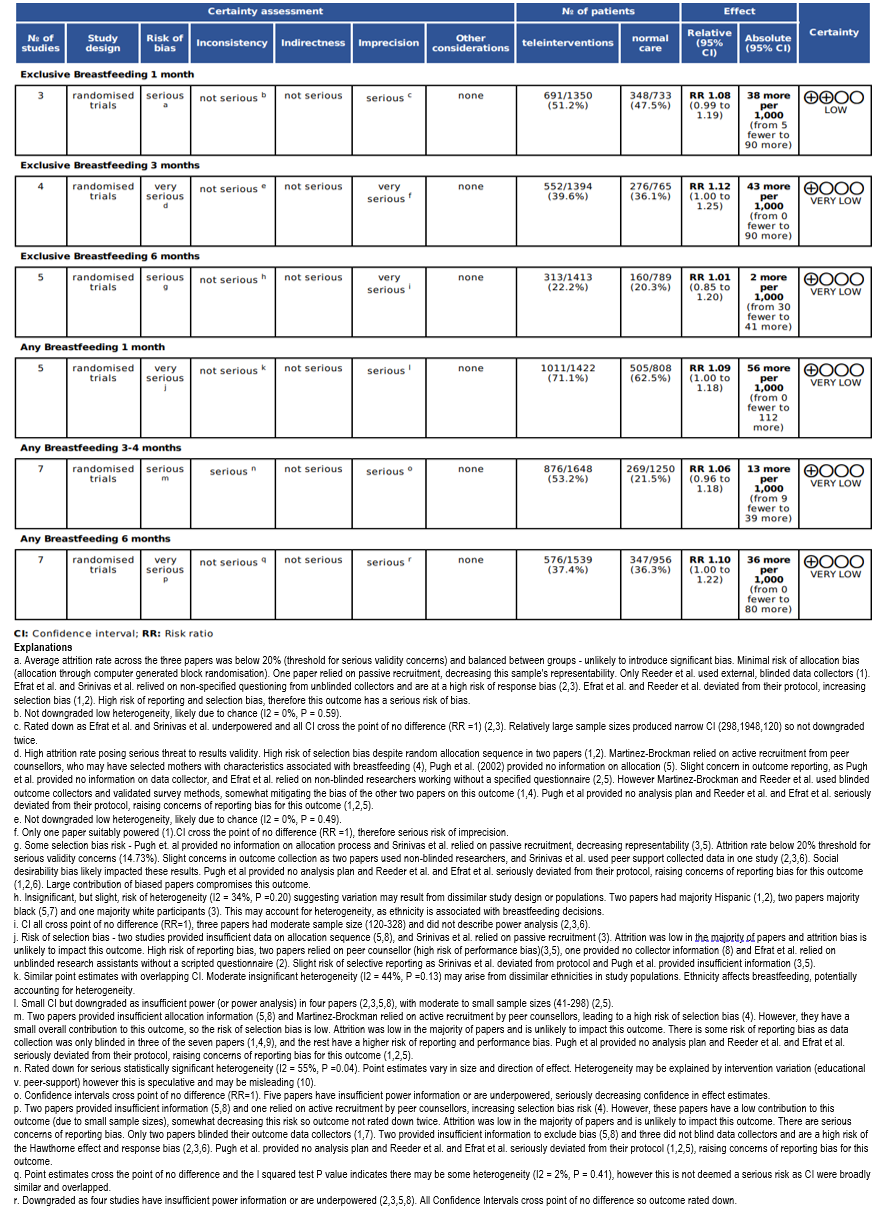
Figure 1 – Grade Summary of Findings Table .jpg
